# Differential transcriptome study on the damage of testicular tissues caused by chronic infection of *T. gondii* in mice

**DOI:** 10.1186/s13071-024-06247-z

**Published:** 2024-06-10

**Authors:** Haoxin Li, Hao Yuan, Zi-Peng Yang, Yining Song, Jun-Jie Wang, Qingyuan Wen, Yu-Xiang Zheng, Xiu-Xiang Zhang, Miao Yu, Zi-Guo Yuan

**Affiliations:** 1 Key Laboratory of Zoonosis Prevention and Control of Guangdong Province, College of Veterinary Medicine, South China Agricultural University, Guangzhou, 510642 Guangdong People’s Republic of China; 2 College of Plant, South China Agricultural University, Guangzhou, 510642 Guangdong People’s Republic of China; 3 Key Laboratory of Oral Medicine, Guangzhou Institute of Oral Disease, Stomatology Hospital of Guangzhou Medical University, Guangzhou, 510140 People’s Republic of China

**Keywords:** *T. gondii* chronic infection, RNA-seq, Testis, BTB

## Abstract

**Background:**

*Toxoplasma gondii* is an intracellular protozoan parasite that is widely distributed in humans and warm-blooded animals. *T. gondii* chronic infections can cause toxoplasmic encephalopathy, adverse pregnancy, and male reproductive disorders. In male reproduction, the main function of the testis is to provide a stable place for spermatogenesis and immunological protection. The disorders affecting testis tissue encompass abnormalities in the germ cell cycle, spermatogenic retardation, or complete cessation of sperm development. However, the mechanisms of interaction between *T. gondii* and the reproductive system is unclear. The aims were to study the expression levels of genes related to spermatogenesis, following *T. gondii* infection, in mouse testicular tissue.

**Methods:**

RNA-seq sequencing was carried out on mouse testicular tissues from mice infected or uninfected with the *T. gondii* type II Prugniaud (PRU) strain and validated in combination with real-time quantitative PCR and immunofluorescence assays.

**Results:**

The results showed that there were 250 significant differentially expressed genes (DEGs) (*P* < 0.05, |log_2_fold change| ≧ 1). Bioinformatics analysis showed that 101 DEGs were annotated to the 1696 gene ontology (GO) term. While there was a higher number of DEGs in the biological process classification as a whole, the GO enrichment revealed a significant presence of DEGs in the cellular component classification. The Arhgap18 and Syne1 genes undergo regulatory changes following *T. gondii* infection, and both were involved in shaping the cytoskeleton of the blood–testis barrier (BTB). The number of DEGs enriched in the MAPK signaling pathway, the ERK1/2 signaling pathway, and the JNK signaling pathway were significant. The PTGDS gene is located in the Arachidonic acid metabolism pathway, which plays an important role in the formation and maintenance of BTB in the testis. The expression of PTGDS is downregulated subsequent to *T. gondii* infection, potentially exerting deleterious effects on the integrity of the BTB and the spermatogenic microenvironment within the testes.

**Conclusions:**

Overall, our research provides in-depth insights into how chronic *T. gondii* infection might affect testicular tissue and potentially impact male fertility. These findings offer a new perspective on the impact of *T. gondii* infection on the male reproductive system.

**Graphical Abstract:**

**Figure:**
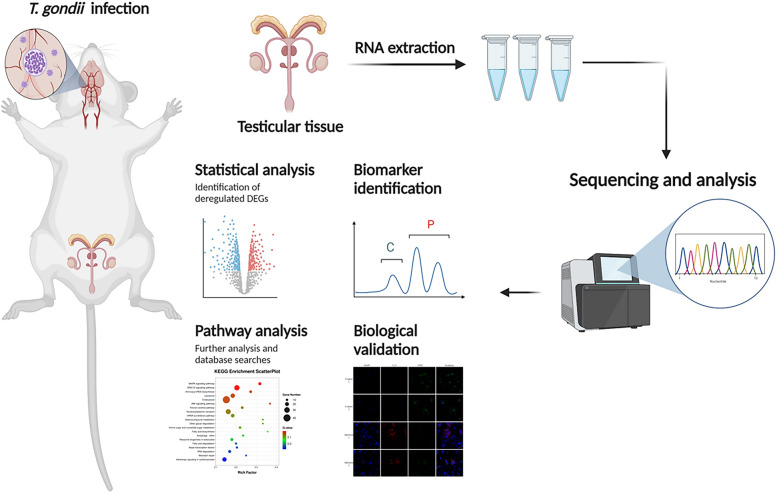

## Background

*Toxoplasma gondii* (*T. gondii*) is an intracellular protozoon parasite of significant zoonotic importance [1]. *T. gondii* infection can traverse the blood–brain barrier (BBB), leading to *T. gondii* encephalopathy [2, 3]. In pregnant women, the *T. gondii* infection can be vertically transmitted to the fetus, resulting in miscarriage, stillbirth, and abnormalities [4]. However, *T. gondii* infection is often chronic and asymptomatic.

Previous studies investigated the *T. gondii* infection on male reproductive health for understanding and addressing potential causes of male reproductive disorders. Recently, it was reported that *T. gondii* infection affects the synthesis and secretion of testosterone in the Leydig cells [5], damages germ cell structure [6], and even changes the cell apoptosis [7–9]. Moreover, it negatively affects the sperm parameters [10], which would directly or indirectly affect spermatogenesis in the testis. To the best of our knowledge, there is no research on the mechanism of male reproductive failure caused by *T. gondii* infection.

The genotypes of *T. gondii* were divided into three types I, II, and III. Notably, the prevalent type II genotype in human *T. gondii* infections is associated with relatively weak pathogenicity and the formation of brain cysts [11]. In our study, mice infected with *T. gondii* Prugniaud (PRU) strain were sequenced and identified by RNA-seq in testis. The significant differentially expressed genes (DEGs) before and after *T. gondii* infection were analyzed to provide a reference for understanding the regulation mechanisms of *T. gondii* infection in male reproductive disorders.

## Methods

### Sample collection

Six 8-week-old Kunming (KM) male mice were purchased from the laboratory animal center of Guangdong Province, China. They were divided equally into two groups: three mice in the experimental group and three in the control group. In the experimental group, *T. gondii* PRU strains were intragastrically administered into the mice, which were loaded with four cysts (average 30 μm diameter). On the day 35 post-infection, the mice were sacrificed by cervical dislocation following the standard ethical regulations. The testicular tissues were quickly harvested in a sterile environment and the surrounding adipose tissues and blood vessels were separated under a stereo microscope and placed in an RNase-free centrifuge tube in liquid nitrogen. All samples were stored at −80 °C.

### Total RNA extraction and analysis of RNA-seq

Trizol method was used to extract total RNA from testicular tissues of KM mice. The quality and purity of the extracted total RNA were detected using an Agilent 2100 Bioanalyzer and RNA6000 Nano LabChip Kit (Agilent, CA, USA). Sequencing libraries were constructed using the AM Pure XP beads method on the detected total RNA using Illumina Hiseq2500. To guarantee the quality of information analysis, raw reads have been sequenced and filtered to get clean reads. At the same time, the sequence repeatability of Q20, Q30, GC content, and clean reads was calculated, and all downstream analyses were based on high-quality clean reads. Using the Trinity program, clean reads were spliced to obtain transcript sequences, and the longest transcript in each gene was taken as unigene for subsequent analysis.

### Identification and annotation of DEGs

To obtain comprehensive gene function information, clean reads were compared with five databases for gene function annotation and compared with the clean reads of a certain gene, which was only compared with the reference genes (unique match) in at least one pair, and was defined as an expression gene. Functional annotation of sequences was based on the following databases: SwissProt protein sequence database, non-redundant protein database, Kyoto Encyclopedia of Genes and Genomes (KEGG), Karyotic Orthologous Group database, and Pfam. Gene expression levels were measured by the Reads Per Kilobase of exon model per million mapped reads (RPKM) value. The RPKM value of each gene was calculated using the MA-plot-based method with random sampling model (MARS) model in the DEGseq program. If the RPKM value was greater than 1000, it was considered to be a highly expressed gene. We set a *P* value of < 0.05 and |log_2_fold change| ≥ 1” as the threshold for judging DEGs. The functional enrichment and classification of DEGs were carried out in the gene ontology (GO) and KEGG database and the *P* value < 0.05 was used as a threshold to determine the significant enrichment of GO term or pathway.

### RT-qPCR and western blotting analysis

Real-Time Quantitative and western blotting analysis to verify the expression level of mRNA. Premier 5 software (Premier Biosoft International, Palo Alto, CA, USA) was used to design real-time quantitative PCR (qPCR) specificity primers for DEGs. The genes were based on genes closely related to reproduction, and *β*-actin was an internal reference gene. The specificity detection primers of qPCR and their length are presented in Table 1. The qPCR assay was performed on a Rotor-Gene Q (Qiagen) on the basis of the SYBR Green dye method. The qPCR reaction system (20 μL) protocol was as follows: 10 μL SYBR Premix Ex *Taq* II, ddH_2_O 6 μL, 1 μL each of the upstream and downstream primers (10 μmol/L), and 2 μL of the template (the cDNA concentration was uniformly diluted to 40 ng/μL). The procedures of qPCR reaction include pre-denaturation at 95 °C for 5 min, denaturation at 95 °C for 30 s, and annealing at 60 °C for 1 min for 40 cycles. The procedures were repeated three times for each sample. The relative expression of each gene was calculated using the 2^–ΔΔCt^ method.

**Table 1:** RNA extract quality results

Sample	Conc. (µg/µL)	O.D. 260/280	O.D. 260/230	Amount (µg)	rRNA	RIN	QC evaluation
Tes_PRU1	1.98	2.10	2.32	89.25	1.7	8.5	A
Tes_PRU2	1.68	1.98	2.33	75.6	1.8	7.8	A
Tes_PRU3	1.52	2.04	2.27	68.4	1.9	8.2	A
Tes_Con1	1.26	2.08	2.29	56.65	1.8	8.8	A
Tes_Con2	1.86	2.08	2.08	83.79	2.1	8.7	A
Tes_Con3	1.16	2.06	2.34	52.24	1.9	8.7	A

The transcriptome data were validated through western blot analysis. Two key genes (*PTGDS*, *SYNE1*), and tight junction proteins (Claudin-11 and ZO-1), were chosen for validating their expression levels. Similarly, with *β*-actin as endogenous control, the preprocessed protein sample underwent separation on a 10% Sodium Dodecyl Sulfate Polyacrylamide Gelelectropheresis at 120 V. The proteins were incubated with antibodies including rabbit anti-PTGDS (Abcam, 1:1000), rabbit anti-SYNE1 (Invitrogen, 1:800), rabbit anti-Claudin-11 (Invitrogen, 1:250), and rabbit anti-ZO-1 (Invitrogen, 1:100). Horseradish Peroxidase-conjugated goat anti-rabbit immunoglobulin G (IgG) was used as a secondary antibody at 1:1000. The membrane was visualized using a diaminobenzidine (DAB) substrate solution, and the image was analyzed using western blot detection system.

### Indirect immunofluorescence to detect the expression level of PTGDS protein

The PRU strain of *T. gondii* was subcultured in the mice in our laboratory. The Specific Pathogen Free adult KM mice were sacrificed by cervical dislocation. The testis was removed from the aseptic environment. The tunica albuginea of testis was removed after rinsing with Phosphate Belanced Solution. Type I collagenase was added and digested in a 37 °C water bath for 10 min. DMEM/F-12 culture was added. The liquid was terminated digestion, centrifuged at 1500 rpm for 4 min, the supernatant was discarded, resuspended by adding DMEM/F12 culture solution, filtered through a 200-mesh sieve, centrifuged at 1500 rpm for 4 min, and the supernatant was discarded. DMEM/F-12 medium was added to dilute the cell pellet to obtain a cell suspension. The cells were inoculated into T25 cell culture flasks (containing 10% fetal bovine serum and 1% penicillin–streptomycin), changed for 12 h. After 24 h the cells were scraped off with a cell scraper and inoculated on a 24-cell plate (specific cell-climbing tablet 80% cell density), and after the cells had re-adhered on the slides, they were infected with tachyzoites. When the tachyzoites invaded the mesenchymal cells and formed parasitophorous vacuoles, the culture medium was discarded, fixed in 4% paraformaldehyde for 10 min, then washed three times with PBS for 5 min each time. Finally, 0.2% Triton X-100 was used for 5 min at room temperature. The cells were immersed in PBS thrice for 5 min each. The rabbit serum was blocked at 37 °C for 1 h and washed in PBS three times for 5 min each. Then, the mice anti-PTGDS (1:250) and pig anti-*T. gondii* multi-antisera (1:200) were given as 1:1 added to the wells, left at 37 °C for 30 min, incubated at 4 °C overnight (16 ~ 18 h), rewarmed at 37 °C for 30 min, and washed three times with PBS for 5 min each time. The rabbits anti-porcine Cy3-IgG ((1):500) and rabbit anti-mouse FITC-IgG (1:200) fluorescent secondary antibodies were added to the wells at a ratio of 1:1, and then were incubated at 37 °C in the dark for 1 h, followed by washing three times with PBS for 5 min each time. 4',6-diamidino-2-phenylindole was added to protect the cells from light and incubated for 5 min then washed three times with PBS for 3 min each time. The cells were removed and the slides were mounted with a mounting fluid containing an anti-fluorescence quencher. The images were then observed and captured under a confocal laser microscope.

## Results

### The results of RNA extraction

After the total RNA of the sample was extracted, the quality of the RNA solution was tested. The index of RNA used for the sequencing of the transcriptional group was as follows: Optical Density. 260/280 > 1.8, O.D. 260/230 > 1.5. RNA integrity number (RIN) was used to evaluate RNA integrity, with a full score of 10. Quality control evaluation index refers to the comprehensive evaluation of RIN value, rRNA 28S/18S and O.D. 260/280 ratio, a grade in line with the quality requirements. It shows that RNA has good integrity and meets the requirements of subsequent experiments. The results are presented in Table 1.

### Pretreatment results of sequencing data

The total RNA extracted from testicular tissue samples was used to construct an RNA library and sequenced by Illumina Hiseq2500, and sequencing results are presented in Table 2. About 50.8G of raw reads are obtained in the testicular group. After filtering out the unavailable reads, there are clean reads of about 50.06G, and the sequencing data for each sample is above 6G. More than 98% of the data in the experimental and control groups are valid data, which can be further analyzed.

**Table 2:** Pretreatment results of data quality

Sample	Raw data read	Base	Valid data read	Base	Valid read %	Q20%	Q30%	GC%
Tes_PRU1	52576294	7.91G	52169538	7.83G	98.89	99.04	91.76	49.5
Tes_PRU2	53151742	7.97G	51998349	7.8G	97.83	98.19	91.77	49.3
Tes_PRU3	61921170	9.3G	60806588	9.12G	98.2	99.36	91.15	47.5
Tes_Con1	55115974	8.27G	54548470	8.18G	98.97	98.71	90.92	49
Tes_Con2	56251678	8.4G	55469779	8.33G	98.61	98.42	90.85	49.8
Tes_Con3	59617181	8.95G	58705038	8.80G	98.47	98.66	90.52	49.3

Raw data/read: raw data are sequenced, and the number of sequencing sequences of each file is counted in four units. Raw data/base: the number of sequence sequences multiplied by the length of the sequencing sequence and expressed in unit G. Valid data/read: after preprocessing, the number of sequencing sequences for each file is counted by a unit of four behavior. Valid data/base: after pretreatment, the number of sequence sequences multiplied by the length of the sequencing sequence and expressed in unit G. Valid ratio%: the ratio of processed data (valid) to original data (raw) is expressed as percentage. Q20%: error rate is 1%; Q30%: error rate is 0.1%. GC count%: the GC content of the data in the original data

### RNA‑sequencing data analysis

**Fig. 1:**
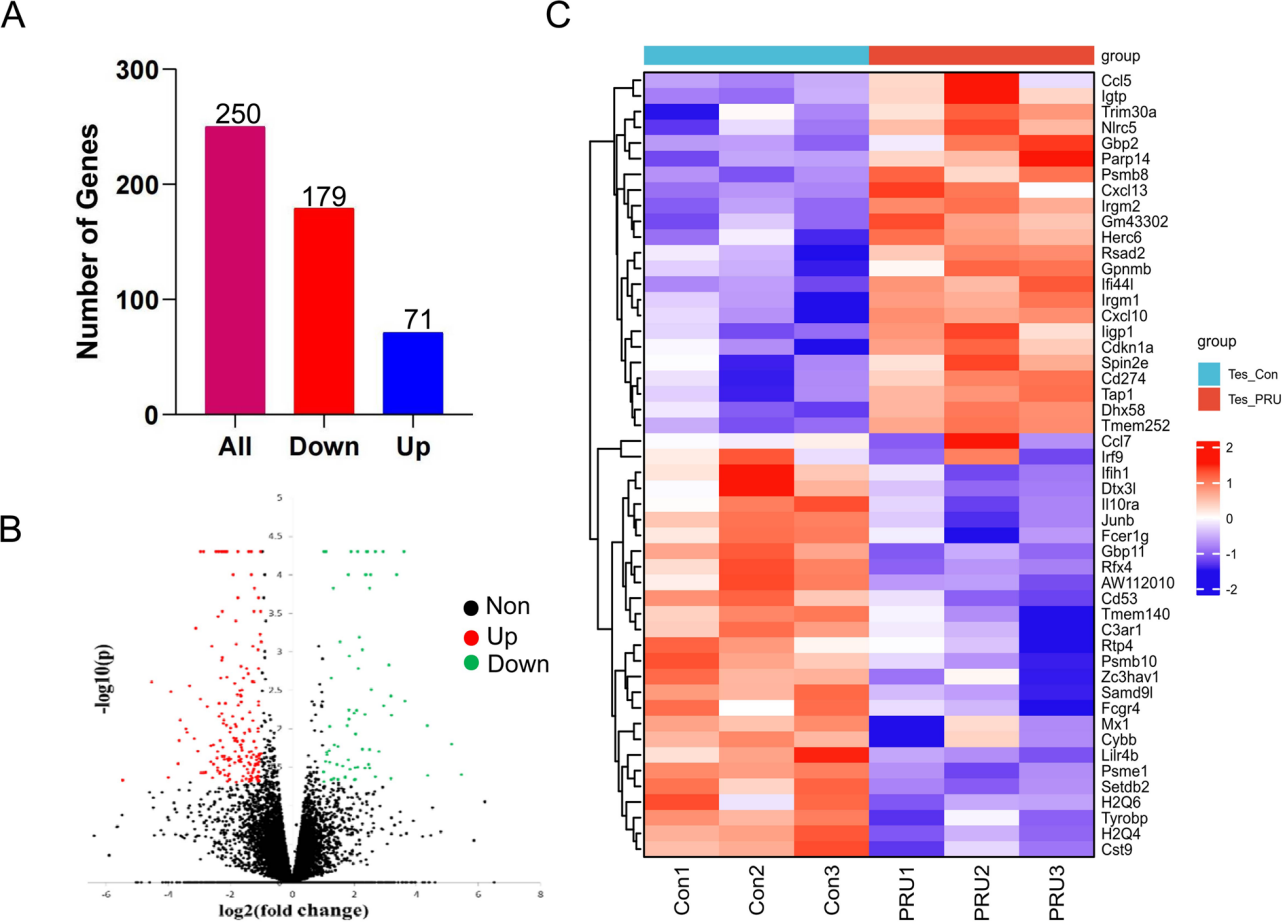
RNA‑sequencing analysis revealing the regulatory changes in testicular tissue post-*T. gondii* infection. **A** Shows the total number of differentially expressed genes, with the number of genes downregulated (179) shown in red and the number of genes upregulated (71) shown in blue, out of the total (250) shown in pink. **B** A volcano plot where the *x*-axis represents the log_2_ fold change and the *y*-axis represents the negative logarithm of the *P*-value [−log_10_(*P*-value)]. Points above the threshold lines represent significantly differentially expressed genes, with upregulated genes in red, downregulated genes in green, and non-significant genes in black. **C** A heat map displaying the expression levels of top 50 DEGs across different samples or conditions. Red indicates higher expression, and blue indicates lower expression. The groups Tes_Con and Tes_PRU represent control and experimental groups, respectively

RNA-seq technique was used to sequence the testicular tissues of the *T. gondii* infection and control groups. The results show that there are a total of 250 genes at the level of expression (*P* < 0.05, |log_2_ fold change| > 1), including 179 downregulated and 71 upregulated genes (Fig. 1A). We drew a volcano map to observe these differences more intuitively (Fig. 1B). The expression levels of top 50 DEGs in the experimental and control groups were observed by thermography (Fig. 1C).

The data showed significant differences between the infected group and the control group, which also proved the reliability of the data.

### KEGG and GO enrichment analysis

To better understand the biological functions of DEGs, we performed bioinformatics analysis on them. We conducted a comprehensive KEGG pathway including six modules (cellular processes, environmental information processing, genetic information processing, human diseases, metabolism, organismal systems).

**Fig. 2:**
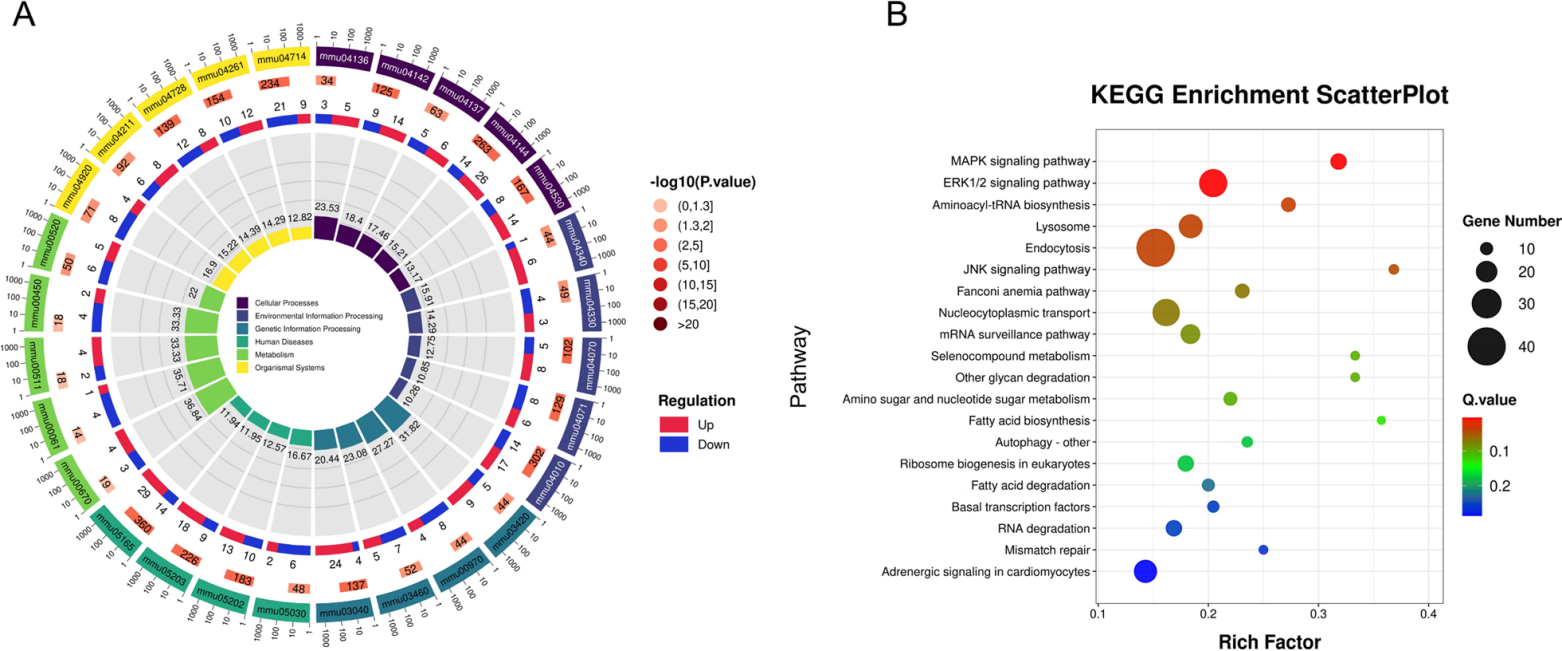
DEG KEGG pathway analysis. **A** DEG circos plot. Particular plot is color-coded to show different categories such as cellular processes, environmental information processing, etc., with each segment representing a KEGG pathway. The width of each segment is proportional to the number of genes involved. The inner rings show the level of gene regulation (up in red, down in blue) and the significance of enrichment [−log_10_(*P*-value)]. **B** KEGG enrichment scatterPlot. The *x*-axis is the rich factor, which is a measure of the enrichment level, while the *y*-axis indicates the specific pathways. The size of each dot correlates with the number of genes (gene number) involved, and the color indicates the Q.value (adjusted *P*-value), with red being more significant

We found that the DEGs were primarily enriched in the modules of cellular processes, genetic information processing, and metabolism (Fig. 2A). Further analysis revealed significant enrichment of DEGs in the endocytosis signaling pathway. Additionally, DEG regulation was most notable in the MAPK signaling pathway, the ERK1/2 signaling pathway, the Aminoacyl-tRNA biosynthesis signaling pathway, and the JNK signaling pathway (Fig. 2B).

It is noteworthy that the MAPK signaling transduction system is involved in cell proliferation, differentiation, apoptosis, and responses to environmental stimuli. In the testes of mammals, MAPK can indirectly affect the development of germ cells by influencing the function of supporting cells, such as Sertoli cells [12]. Additionally, the ERK1/2 and JNK signaling pathways also play a significant role in testicular damage. The data suggest that the activation of the MAPK, ERK1/2, and JNK signaling pathways caused by *T. gondii* may be related to testicular tissue damage, including inflammation, cell death, and interference in the process of spermatogenesis.

**Fig. 3:**
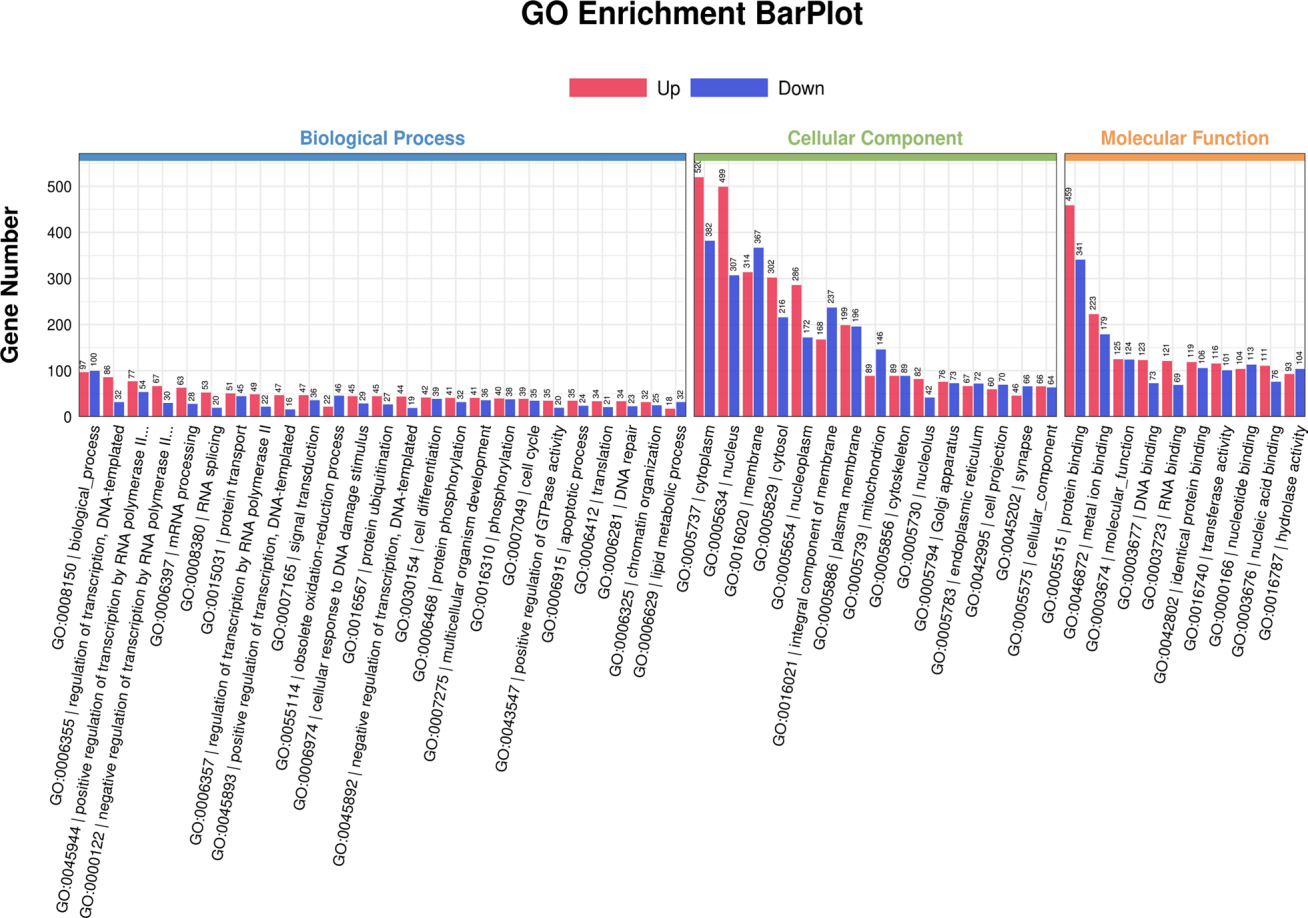
DEG GO analysis. Red bars represent the number of genes upregulated, while blue bars represent downregulated genes. The height of each bar corresponds to the number of genes associated with each GO term. The *x*-axis represents the GO term, and the *y*-axis represents the number of genes

The GO classification is divided into three major categories, including cellular component, molecular function, and biological process. The number of DEGs is labeled in Fig. 3.

In the biological processes category, significant changes are observed in gene expression related to gene transcription, RNA metabolism, cell cycle, and apoptosis. These changes may indicate that post-infection, cells are attempting to regulate these fundamental biological processes to combat the pathogen. In the cellular component category, differentially expressed genes are mainly enriched in organelles, membranes, and protein complexes, which may relate to the impact of *T. gondii* infection on cell structure and function. In the Molecular Function category, changes in protein binding functions and Adenosine Triphosphate binding are observed, which might be associated with alterations in cellular energy metabolism and signal transduction. These data may indicate the extensive impact of *T. gondii* infection on testicular tissue, including but not limited to cell death, reproductive dysfunction, and inflammatory responses.

### RT-qPCR and western blotting verification results

**Fig. 4:**
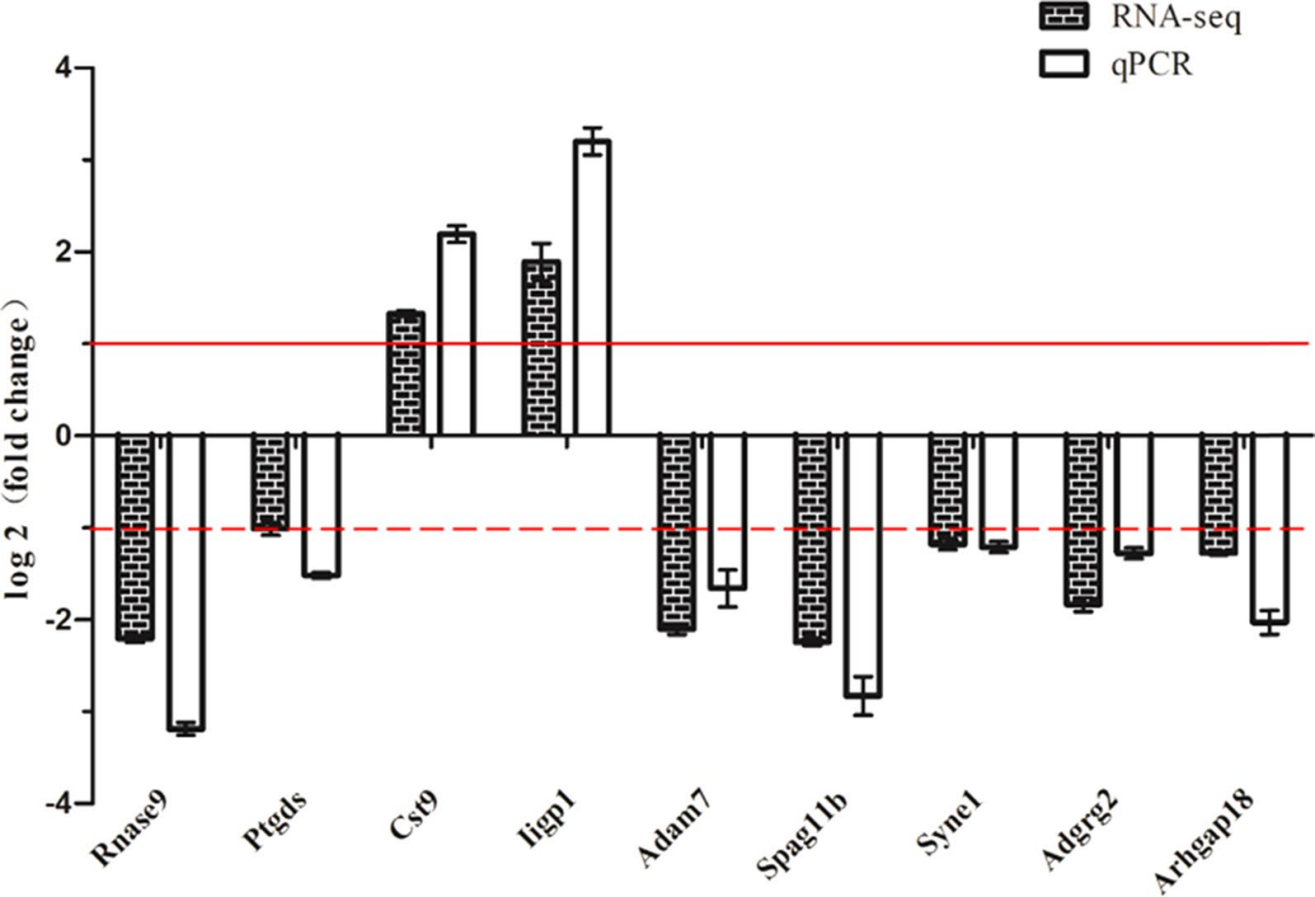
Comparison diagram of qPCR analysis and RNA-seq. In the picture, the solid line shows log_2_ (fold change) = 1, greater than or equal to 1 shows the gene significant upregulated expression; the dotted line shows log_2_ (fold change) = −1, less than or equal to −1 shows the gene significant downregulated expression

We selected nine DEGs closely related to the reproductive system (Table 3). qPCR specificity primers for these DEGs were designed using Premier 5 software (Premier Biosoft International, Palo Alto, CA, USA) (Table 4). The nine key genes related to reproduction were verified by qPCR, and the relative expression level were compared with the RNA-seq data. The results show that the expression trends of these genes align with the RNA-seq results, as shown in (Fig. 4).

**Table 3:** Parts of DEGs related to reproduction

Gene name	log_2_fold change	Function
Rnase9	−2.20757	Regulate sperm motility, participate in sperm capacitation, lack of sperm disorders will lead to maturity
Ptgds	−1.01194	Expressed in testis and epididymis cells and secreted substances into semen, regulating prostaglandin biosynthesis, participating in the development and maintenance of the BTB, and playing an important role in the maturation and maintenance of the male reproductive system
Cst9	1.32794	Development of testis is expressed in the process of supporting cells forming testicular cord
Iigp1	1.89148	Resistance to intracellular pathogens can destroy the intracellular vacuoles of *T. gondii* and kill and kill *T. gondii*
Adam7	−2.09991	Plays an important role in male reproduction, including sperm maturation and gonadal function
Spag11b	−2.24253	Has the antibacterial activity of beta defensin and its unique reproductive function, such as promoting sperm maturation and obtaining, improving sperm motility, and crossing zona pellucida
Syne1	−1.18019	Related to the cytoskeletal structure of spermatozoa and plays an important role in the process of nuclear remodeling in the sperm head
Adgrg2	−1.83472	Participates in signal transduction pathway to control epididymal function and male fertility
Arhgap18	−1.27599	Regulates cell shape, spreading, and migration

**Table 4:** qPCR specific primers and product length

Gene name	Forward primer	Reverse primer	Product length
Rnase9	GAGTACAGGGCCCACCAAAC	ACAGCGCCCCTTATAGTGAA	136 bp
Ptgds	CGGCCTCAATCTCACCTCTAC	CCACTGACACGGAGTGGATG	137 bp
Cst9	GAGGCTTCAGCTGCGTAGA	GGTACCGACAGTAAACAGGCA	118 bp
Iigp1	AACTGGGGTGGTGGAGGTAA	CCAGGTAAGTGTTTGGTGGGA	122 bp
Adam7	ATGTGTGATGGGCGATGGAA	TTCGGGAAAGGGGTTGTTGA	121 bp
Spag11b	TACCACGAGCCTGAACCAAA	AACGGATGTAAGCAGCAGGG	137 bp
Syne1	TCAGCAGTCTGTGACGGTTC	ACGACTTGAGGGCAGACTTG	107 bp
Adgrg2	TTCTTTGAAACACCCGCCCT	AGTGCGACTGTCACGTTTCT	128 bp
Arhgap18	CTCAGCCAAGAAAGTGGGGT	CTCGGCATTCGGGTTCAGTT	141 bp

**Fig. 5:**
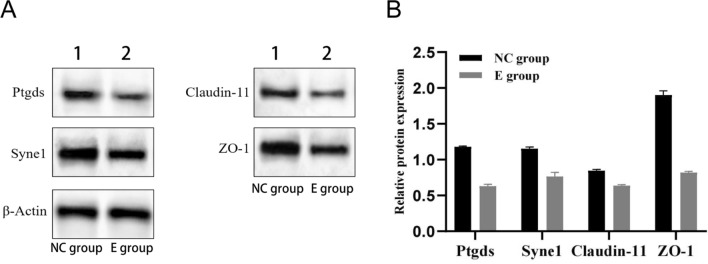
Western blot analysis of the expression levels of Ptgds, Syne1, Claudin-11, and ZO-1. **A** Lane 1 shows the data from the control group. Lane 2 shows the data from the experimental group; *β*-actin is the endogenous control. **B** Quantification of the results shown in panel A using Gel-Pro Analyzer 4.0 software

*T. gondii* infection in testicular tissue induces the aberrant expression of PTGDS and SYNE1 in the host organism. As shown in Fig. 5, *T. gondii* infection in testicular tissue also leads to a decrease in the expression levels of PTGDS and SYNE1 within the host organism. We concurrently assessed the tight junction proteins Claudin-11 and ZO-1 expression levels in the BTB. The experimental results demonstrate a decrease in the expression levels of Claudin-11 and ZO-1 following *T. gondii* infection in testicular tissue (Fig. 5).

### Protein expression level of PTGDS in Leydig cells

**Fig. 6:**
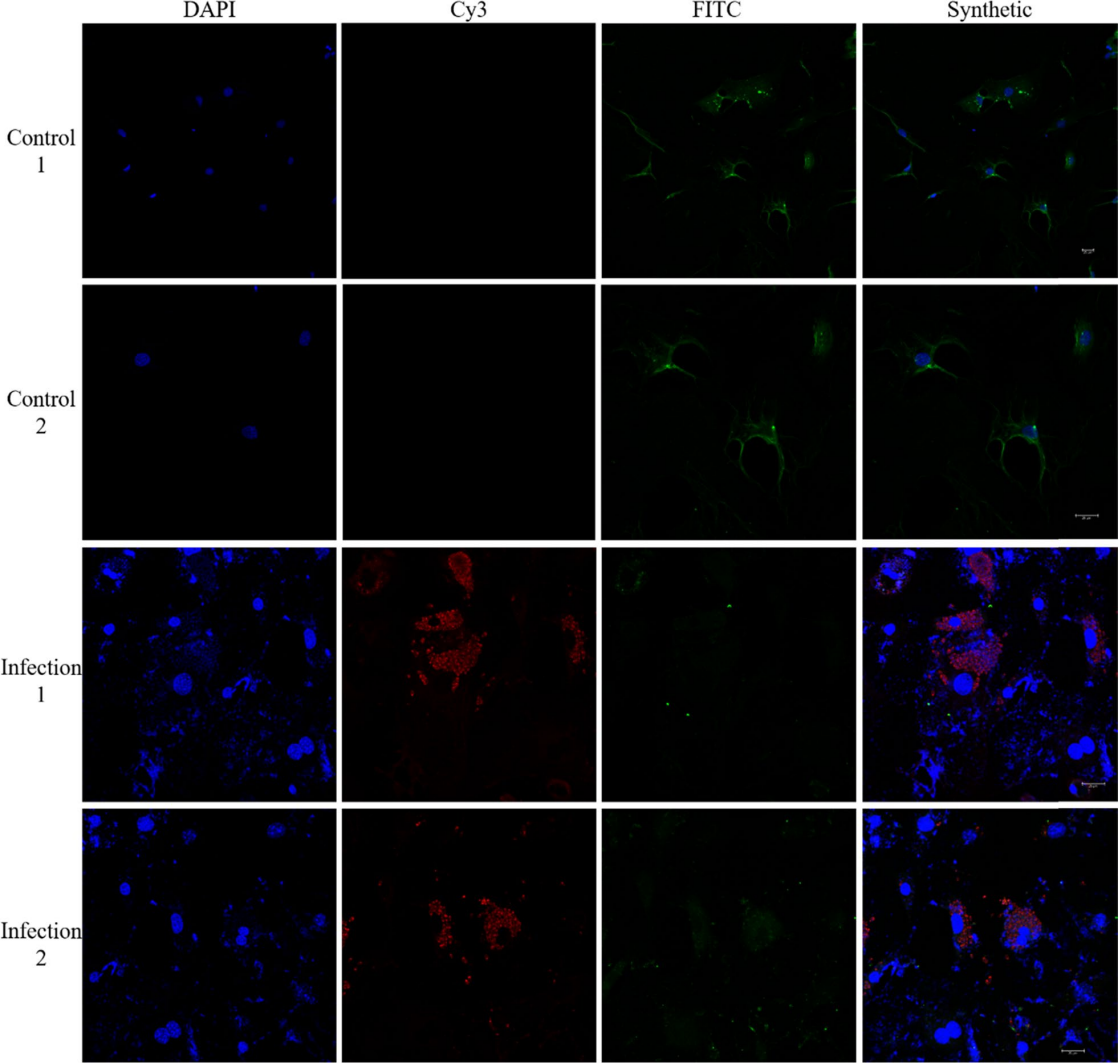
Immunofluorescence of PTGDS expression in Leydig cells infected with tachyzoite of *T. gondii*. In the picture, the blue is the nucleus of DAPI staining, the red is *T. gondii* protein labeled by Cy3, and the green is the PTGDS protein labeled by FITC; control 1, 2, infection 1, 2 are the parallel tests

The results of cell immunofluorescence showed that the protein expression of PTGDS gene in Leydig cells decrease significantly in the experimental group compared with the control group. The DAPI nucleation is found in the *T. gondii* infection group, indicating the phenomenon of nuclear rupture and nuclear dissolution of Leydig cells, as shown in Fig. 6. The results indicate that *T. gondii* infection significantly alters the expression of PTGDS gene in host cells.

## Discussion

### PTGDS and BTB

The arachidonic acid (AA) metabolism pathway is the primary way to generate inflammatory mediators in various tissues [13]. It is an essential fatty acid of human body, widely distributed in the body. AA is converted mainly through three ways: prostaglandins (PGs), thromboxanes (TXs), and lipoxygenase (LOX), which catalyze the production of leukotrienes (LTs) and oxidized 20-carbon enoic acid (epoxyeicosatrienoic) through the COX C-acid, EET, and cytochrome P450 (CYP450) pathway [13–15].

Prostaglandin D2 synthase (PTGDS) is the main component of the body tissue barrier [16], which participates in the formation and maintenance of blood brain, blood retina, blood aqueous humor, and BTB. It plays a crucial role in the maturation and maintenance of the the central nervous and male reproductive systems. In the reproductive system, PTGDS is expressed in the testis (stromal cells, support cells, and spermatogenic cells) and the epididymis (sperm and epididymal epithelial cells), with the ability to be secreted in the semen [17].

In our previous research, we conducted RNA-seq analysis on the epididymal tissues of mice infected with *T. gondii*. Our results revealed an upregulation of PTGDS (log_2_fold change = 0.803089) [18]. However, in this study, the RNA-seq sequencing data demonstrated a significant downregulation of PTGDS in the testicular tissue (log_2_fold change = −1.01194). This finding suggests that after *T. gondii* infection, there is a decrease in PTGDS expression within the testicular tissue, which may indicate inhibitory immune regulation, while an increase was observed in its expression within the epididymal tissue, suggesting activation of the immune response.

In the molecular functional classification of GO, there are functions of PTGDS processes, including binding with fatty acids, participation in intracellular and extracellular participation in the lipid metabolism process, and in the biological process.

The expression of PTGDS in the testicles significantly increases during puberty, and during this period, it coincides with the development of the blood–testosterone barrier. In in vitro experiments, when the specific intercellular connection between cells and stem cells appeared, the expression of PTGDS increased. However, the expression of PTGDS mRNA was expressed by adding trypsin destruction to the intercellular connection in the culture medium [19]. Similar expression patterns of PTGDS are observed in the blood–brain and blood–retina barriers [17]. Given that the support cells are critical components of the BTB, PTGDS likely play a role in the forming and maintaining of blood tissue barrier. Downregulation of PTGDS may have a detrimental impact on the blood–testosterone barrier.

PTGDS is also expressed in the Leydig cells between the seminiferous tubules. Leydig cells promote the development of reproductive organs by secreting testosterone, maintaining secondary sex characteristics, and promoting spermatogenesis. Androgens interact with the androgen receptor on the support cells, Leydig cells, and neoplastic cells [20], establishing a microenvironment conducive to spermatogenesis. This is crucial for maintaining normal spermatogenesis in the testis.

Previous research reported a significant reduction in serum testosterone levels in the male rats infected with *T. gondii*. It was found that *T. gondii* could affect the synthesis and secretion of testosterone in stromal cells [21]. When the ethane two methane sulfonate (EDS) treatment caused the destruction of Leydig cells in the rat testis, the synthesis of androgen was blocked, and the level of androgen receptor in a variety of cells in the testicles decreased, affecting the occurrence of sperm. After exogenous testosterone replacement therapy, the level of androgen receptor was restored [22], which explains the importance of normal secretion of testosterone from stromal cells to spermatogenesis. After selective removal of testosterone with EDS, Boekelheide and colleagues found interstitial cell deletion, cytoplasmic vacuolization of the corresponding segments, fragmentation of nuclei, and degradation [23], indicating that testosterone deficiency destroys the structural integrity of supporting cells and destroys BTB. At the same time, other studies have found that androgen can be used as a regulator of blood testosterone barrier cells to maintain BTB integrity and temporary dissociation and reconstruction [24]. It indicates that testosterone secreted by Leydig cells plays a protective role in BTB.

Previous research done by Garza and his team indicates that the increase of mRNA and protein expression levels of PTGDS is the main cause of male testosterone loss [25]. This subsequently reflects that PTGDS is associated with the level of testosterone, and the increase of PTGDS can cause the increase of the level of testosterone in the synthesis and secretion of Leydig cells. In this experiment, the results of RNA-seq in the testicular group showed that the level of PTGDS mRNA was down, and the level of testosterone production and secretion of Leydig cells would also decrease, thus damaging the BTB.

In 1975, Olsson and his research team observed a significant decline in PTGDS concentration in the seminal plasma of patients with oligospermia [26]. Meanwhile, other research found that the reason could be associated with the impairment of the spermatogenic duct of the testis [27]. Subsequently, the concentration of PTGDS in seminal plasma was utilized as an auxiliary diagnostic index for oligozoospermia [28].

### Cytoskeleton and BTB

The BTB is composed of the basilar membrane of convoluted tubule, the vascular endothelial basement membrane, connective tissue, and Sertoli cells, to which the basement is closely connected. The junctional complex between the Sertoli cells constitutes the most important part of the BTB [29]. BTB consists of tight junction (TJ) based on actin, basic ectoplasmic specialization (ES), gap junctions (GJ) and filamentous desmosome (DS) between Sertoli cells [30]. Actin microfilament networks in the basal ES contribute to strengthening the structural integrity of TJ [31, 32].

The Cytoskeleton is a network of filamentous structures located in the intracellular, almost involved in various cellular activities. It forms a three-dimensional reticular structure woven with protein fibers, filling the entire cytoplasmic space and establishing structural connections with the cell and nuclear membranes. The cytoskeleton is essential for maintaining the specific shape of cells and is involved in cell movements [33]. Microfilaments are primarily composed of actin, which is related to the movement and connection of cells, so it is also called actin microfilaments. They consist of two forms of actin in Sertoli cells: globular-actin (G-actin) and polymerized fibrin action (F-actin) [34]. Microfilaments provide structural support to Sertoli cells, and contribute to network structure formation within the closely connected system between Sertoli cells [35]. They actively regulate the tight junctions of the blood-testis barrier, facilitating spermatogenic cells from the basal compartment to the abluminal compartment. Therefore, the damage of cytoskeleton of Sertoli cells results in abnormal cell morphology and dysfunction of TJ, which makes the BTB cannot maintain its stability.

In our experiment, several DEGs related to the cytoskeleton were identified in testis tissue, including Ras homologous oncogenes (Rho) GTPase-activating protein 18 (Arhgap18) and Nesprin-1 (Syne1) gene, among others. Rho GTPase-activating protein inhibits the polymerization of F-actin and regulates cell shape, diffusion, and migration. Rho GTPases serve as critical mediators in signal transduction. It is a downstream effector protein of various membrane surface receptors, including G-protein-coupled receptor, tyrosine kinase receptor, cytokine receptor, and adhesion molecule receptor. In the process of cell signal transduction, Rho GTPases play the role of “molecular switch,” quickly converting between the activated state of GTP binding and the non-activated state of GDP binding. They transmit the extracellular signal to the intracellular [36], and affect the morphology and movement of the cells by regulating the reorganization of actin and the cytoskeleton [37]. Rho protein is an important intermediate signal molecule during the process of intracellular signal transduction. The Rho and its downstream effector proteins regulate the cell barrier function and intestinal permeability by maintaining the stable state of TJ and adhesion connections between intestinal epithelial cells [37].

In our study, the expression of the Arhgap18 gene was significantly downregulated, with a log_2_fold change = −1.27599. This downregulation will weaken the inhibition of Rho, indirectly leading to a weakening of the inhibition of F-actin. Consequently, this will result in the strengthening of the polymerization capacity of actin, and the protein network of actin forms the basis of the tight junction of the BTB.

When the cytoskeleton changes, the gap increases between the tight junction when F-actin polymerization increases, leading to the destruction of BTB, breaking the balance between the microenvironment and subsequently male sterility. Syne1 forms a connection network between the cell organs and the actin cytoskeleton to maintain subcellular space tissue, and participates in the connection between the nuclear lamina and the cytoskeleton. In addition, the Syne1 gene may be involved in nuclear remodeling of sperm head formation during spermatogenesis [38, 39]. Compared with the control group, the expression of the Syne1 gene was significantly downregulated, log_2_fold change = −1.18019, which had a negative effect on the formation of BTB and the morphological structure of sperm. The BTB is an effective protective barrier for male reproductive system. The main function is to prevent some large molecular substances from the blood or lymphatic pathways into the convoluted tubule cavity to regulate the concentration of activator substances in the spermatogenic epithelium [40]. In chronic infection of *T. gondii*, the BTB is destroyed and thus disorder of spermatogenesis occurs, then affecting the maturation and capacitation of spermatozoa and decreasing male reproductive capacity.

## Conclusions

Our research reveals the impact of chronic *T. gondii* infection on mouse testicular tissue. *T. gondii* infection resulted in a significant differential expression of genes, with a notable downregulation of PTGDS, a gene involved in prostaglandin synthesis and the maintenance of BTB. The findings imply that *T. gondii* infection may have adverse effects on the integrity of the BTB. This research provides in-depth insights into how chronic *T. gondii* infection might affect testicular tissue and potentially impact male fertility. Our findings could contribute toward development of therapeutic strategies for infection-related male reproductive disorders.

## Data Availability

The transcriptome data generated in this study have been deposited into NCBI BioProject (accession no. PRJNA552423).

## References

[1] Montoya JG, Liesenfeld O. Toxoplasmosis. Lancet. 2004;363:1965–76. 10.1016/s0140-6736(04)16412-x. 15194258 10.1016/S0140-6736(04)16412-X

[2] Feustel SM, Meissner M, Liesenfeld O. *Toxoplasma gondii* and the blood-brain barrier. Virulence. 2012;3:182–92. 10.4161/viru.19004. 22460645 10.4161/viru.19004 PMC3396697

[3] Haroon F, Händel U, Angenstein F, Goldschmidt J, Kreutzmann P, Lison H, Fischer KD, Scheich H, Wetzel W, Schlüter D, Budinger E. *Toxoplasma gondii* actively inhibits neuronal function in chronically infected mice. PloS ONE. 2012;7:e35516. 10.1371/journal.pone.0035516. 22530040 10.1371/journal.pone.0035516 PMC3329480

[4] Megli CJ, Coyne CB. Infections at the maternal-fetal interface: an overview of pathogenesis and defence. Nat Rev Microbiol. 2022;20:67–82. 10.1038/s41579-021-00610-y. 34433930 10.1038/s41579-021-00610-y PMC8386341

[5] Eslamirad Z, Hajihossein R, Ghorbanzadeh B, Alimohammadi M, Mosayebi M, Didehdar M. Effects of *Toxoplasma gondii* infection in level of serum testosterone in males with chronic *Toxoplasmosis*. Iran J Parasitol. 2013;8:622–6. 25516745 PMC4266128

[6] Shukla KK, Mahdi AA, Rajender S. Apoptosis, spermatogenesis and male infertility. Front Biosci. 2012;4:746–54. 10.2741/415. 10.2741/415 22201910

[7] Wang RA, Nakane PK, Koji T. Autonomous cell death of mouse male germ cells during fetal and postnatal period. Biol Reprod. 1998;58:1250–6. 10.1095/biolreprod58.5.1250. 9603260 10.1095/biolreprod58.5.1250

[8] Sinha Hikim AP, Swerdloff RS. Hormonal and genetic control of germ cell apoptosis in the testis. Rev Reprod. 1999;4:38–47. 10.1530/ror.0.0040038. 10051101 10.1530/ror.0.0040038

[9] Kajihara T, Okagaki R, Ishihara O. LPS-induced transient testicular dysfunction accompanied by apoptosis of testicular germ cells in mice. Med Mol Morphol. 2006;39:203–8. 10.1007/s00795-006-0334-7. 17187183 10.1007/s00795-006-0334-7

[10] Colosi HA, Jalali-Zadeh B, Colosi IA, Simon LM, Costache CA. Influence of *Toxoplasma gondii* infection on male fertility: a pilot study on immunocompetent human volunteers. Iran J Parasitol. 2015;10:402–9. 26622295 PMC4662740

[11] Ajzenberg D, Cogné N, Paris L, Bessières MH, Thulliez P, Filisetti D, et al. Genotype of 86 *Toxoplasma gondii* isolates associated with human congenital toxoplasmosis, and correlation with clinical findings. J Infect Dis. 2002;186:684–9. 10.1086/342663. 12195356 10.1086/342663

[12] Wang H, Zhou W, Zhang J, Li H. Role of JNK and ERK1/2 MAPK signaling pathway in testicular injury of rats induced by di-N-butyl-phthalate (DBP). Biol Res. 2019;52:41. 10.1186/s40659-019-0248-1. 31387634 10.1186/s40659-019-0248-1 PMC6685163

[13] Goldyne ME, Burrish GF, Poubelle P, Borgeat P. Arachidonic acid metabolism among human mononuclear leukocytes. Lipoxygenase-related pathways. J Biol Chem. 1984;259:8815–9. 6430891

[14] Kroetz DL, Zeldin DC. Cytochrome P450 pathways of arachidonic acid metabolism. Curr Opin Lipidol. 2002;13:273–83. 10.1097/00041433-200206000-00007. 12045397 10.1097/00041433-200206000-00007

[15] Agarwal S, Achari C, Praveen D, Roy KR, Reddy GV, Reddanna P. Inhibition of 12-LOX and COX-2 reduces the proliferation of human epidermoid carcinoma cells (A431) by modulating the ERK and PI3K-Akt signalling pathways. Exp Dermatol. 2009;18:939–46. 10.1111/j.1600-0625.2009.00874.x. 19558494 10.1111/j.1600-0625.2009.00874.x

[16] Hoffmann A, Conradt HS, Gross G, Nimtz M, Lottspeich F, Wurster U. Purification and chemical characterization of beta-trace protein from human cerebrospinal fluid: its identification as prostaglandin D synthase. J Neurochem. 1993;61:451–6. 10.1111/j.1471-4159.1993.tb02145.x. 8336134 10.1111/j.1471-4159.1993.tb02145.x

[17] Tokugawa Y, Kunishige I, Kubota Y, Shimoya K, Nobunaga T, Kimura T, et al. Lipocalin-type prostaglandin D synthase in human male reproductive organs and seminal plasma. Biol Reprod. 1998;58:600–7. 10.1095/biolreprod58.2.600. 9475419 10.1095/biolreprod58.2.600

[18] Zheng YX, Zhang XX, Hernandez JA, Mahmmod YS, Huang WY, Li GF, et al. Transcriptomic analysis of reproductive damage in the epididymis of male Kunming mice induced by chronic infection of *Toxoplasma gondii* PRU strain. Parasit Vectors. 2019;12:529. 10.1186/s13071-019-3783-2. 31703718 10.1186/s13071-019-3783-2 PMC6839085

[19] Samy ET, Li JC, Grima J, Lee WM, Silvestrini B, Cheng CY. Sertoli cell prostaglandin D2 synthetase is a multifunctional molecule: its expression and regulation. Endocrinology. 2000;141:710–21. 10.1210/endo.141.2.7329. 10650953 10.1210/endo.141.2.7329

[20] Sar M, Lubahn DB, French FS, Wilson EM. Immunohistochemical localization of the androgen receptor in rat and human tissues. Endocrinology. 1990;127:3180–6. 10.1210/endo-127-6-3180. 1701137 10.1210/endo-127-6-3180

[21] Abdoli A, Dalimi A, Movahedin M. Impaired reproductive function of male rats infected with *Toxoplasma gondii*. Andrologia. 2012;44:679–87. 10.1111/j.1439-0272.2011.01249.x. 22098674 10.1111/j.1439-0272.2011.01249.x

[22] Sriraman V, Sairam MR, Rao AJ. Evaluation of relative roles of LH and FSH in regulation of differentiation of Leydig cells using an ethane 1,2-dimethylsulfonate-treated adult rat model. J Endocrinol. 2003;176:151–61. 10.1677/joe.0.1760151. 12525259 10.1677/joe.0.1760151

[23] Boekelheide K, Fleming SL, Johnson KJ, Patel SR, Schoenfeld HA. Role of Sertoli cells in injury-associated testicular germ cell apoptosis. Proc Soc Exp Biol Med. 2000;225:105–15. 10.1046/j.1525-1373.2000.22513.x. 11044252 10.1046/j.1525-1373.2000.22513.x

[24] Yan HH, Mruk DD, Lee WM, Cheng CY. Blood-testis barrier dynamics are regulated by testosterone and cytokines via their differential effects on the kinetics of protein endocytosis and recycling in Sertoli cells. FASEB J. 2008;22:1945–59. 10.1096/fj.06-070342. 18192323 10.1096/fj.06-070342 PMC2804916

[25] Garza LA, Liu Y, Yang Z, Alagesan B, Lawson JA, Norberg SM, et al. Prostaglandin D2 inhibits hair growth and is elevated in bald scalp of men with androgenetic alopecia. Sci Trans Med. 2012;4:126–34. 10.1126/scitranslmed.3003122. 10.1126/scitranslmed.3003122 PMC3319975 22440736

[26] Olsson JE. Correlation between the concentration of beta-trace protein and the number of spermatozoa in human semen. J Reprod Fertil. 1975;42:149–51. 10.1530/jrf.0.0420149. 803284 10.1530/jrf.0.0420149

[27] Diamandis EP, Arnett WP, Foussias G, Pappas H, Ghandi S, Melegos DN, et al. Seminal plasma biochemical markers and their association with semen analysis findings. Urology. 1999;53:596–603. 10.1016/s0090-4295(98)00550-0. 10096390 10.1016/s0090-4295(98)00550-0

[28] Gerena RL, Irikura D, Urade Y, Eguchi N, Chapman DA, Killian GJ. Identification of a fertility-associated protein in bull seminal plasma as lipocalin-type prostaglandin D synthase. Biol Reprod. 1998;58:826–33. 10.1095/biolreprod58.3.826. 9510973 10.1095/biolreprod58.3.826

[29] Mruk DD, Cheng CY. Tight junctions in the testis: new perspectives. Philos Trans R Soc Lond B Biol Sci. 2010;365:1621–35. 10.1098/rstb.2010.0010. 20403874 10.1098/rstb.2010.0010 PMC2871926

[30] Li N, Mruk DD, Cheng CY. Actin binding proteins in blood-testis barrier function. Curr Opin Endocrinol Diabetes Obes. 2015;22:238–47. 10.1097/med.0000000000000155. 25887390 10.1097/MED.0000000000000155 PMC4447325

[31] Setchell BP. Blood-testis barrier, junctional and transport proteins and spermatogenesis. Adv Exp Med Biol. 2008;636:212–33. 10.1007/978-0-387-09597-4_12. 19856170 10.1007/978-0-387-09597-4_12

[32] Cheng CY, Mruk DD. The blood-testis barrier and its implications for male contraception. Pharmacol Rev. 2012;64:16–64. 10.1124/pr.110.002790. 22039149 10.1124/pr.110.002790 PMC3250082

[33] Vogl AW, Vaid KS, Guttman JA. The Sertoli cell cytoskeleton. Adv Exp Med Biol. 2008;636:186–211. 10.1007/978-0-387-09597-4_11. 19856169 10.1007/978-0-387-09597-4_11

[34] Kopera IA, Su L, Bilinska B, Cheng CY, Mruk DD. An in vivo study on adjudin and blood-testis barrier dynamics. Endocrinology. 2009;150:4724–33. 10.1210/en.2008-1779. 19574397 10.1210/en.2008-1779 PMC2754679

[35] Toyama Y, Maekawa M, Yuasa S. Ectoplasmic specializations in the Sertoli cell: new vistas based on genetic defects and testicular toxicology. Anat Sci Int. 2003;78:1–16. 10.1046/j.0022-7722.2003.00034.x. 12680465 10.1046/j.0022-7722.2003.00034.x

[36] Crosas-Molist E, Samain R, Kohlhammer L, Orgaz JL, George SL, Maiques O, et al. Rho GTPase signaling in cancer progression and dissemination. Physiol Rev. 2022;102:455–510. 10.1152/physrev.00045.2020. 34541899 10.1152/physrev.00045.2020

[37] Na RH, Zhu GH, Luo JX, Meng XJ, Cui L, Peng HJ, et al. Enzymatically active Rho and Rac small-GTPases are involved in the establishment of the vacuolar membrane after *Toxoplasma gondii* invasion of host cells. BMC Microbiol. 2013;13:125. 10.1186/1471-2180-13-125. 23721065 10.1186/1471-2180-13-125 PMC3681593

[38] Dawe HR, Adams M, Wheway G, Szymanska K, Logan CV, Noegel AA, et al. Nesprin-2 interacts with meckelin and mediates ciliogenesis via remodelling of the actin cytoskeleton. J Cell Sci. 2009;122:2716–26. 10.1242/jcs.043794. 19596800 10.1242/jcs.043794 PMC2909318

[39] Zhang X, Lei K, Yuan X, Wu X, Zhuang Y, Xu T, et al. SUN1/2 and Syne/Nesprin-1/2 complexes connect centrosome to the nucleus during neurogenesis and neuronal migration in mice. Neuron. 2009;64:173–87. 10.1016/j.neuron.2009.08.018. 19874786 10.1016/j.neuron.2009.08.018 PMC2788510

[40] Pelletier RM, Byers SW. The blood-testis barrier and Sertoli cell junctions: structural considerations. Microsc Res Tech. 1992;20:3–33. 10.1002/jemt.1070200104. 1611148 10.1002/jemt.1070200104

