# Correction: Differential transcriptome study on the damage of testicular tissues caused by chronic infection of *T. gondii* in mice

**DOI:** 10.1186/s13071-024-06368-5

**Published:** 2024-07-09

**Authors:** Haoxin Li, Hao Yuan, Zi-Peng Yang, Yining Song, Jun-Jie Wang, Qingyuan Wen, Yu-Xiang Zheng, Xiu-Xiang Zhang, Miao Yu, Zi-Guo Yuan

**Affiliations:** 1 Key Laboratory of Zoonosis Prevention and Control of Guangdong Province, College of Veterinary Medicine, South China Agricultural University, Guangzhou, 510642 Guangdong People’s Republic of China; 2 College of Plant, South China Agricultural University, Guangzhou, 510642 Guangdong People’s Republic of China; 3 Department of Periodontics, School and Hospital of Stomatology, Guangdong Engineering Research Center of Oral Restoration and Reconstruction, Guangzhou Key Laboratory of Basic and Applied Research of Oral Regenerative Medicine, Guangzhou Medical University, Guangzhou, 510182 People’s Republic of China

**Correction: Parasites & Vectors (2024) 17:252** 10.1186/s13071-024-06247-z

Following publication of the original article [1], the authors flagged that the corresponding authorship of the article was incorrect, and that the affiliations information of the author Miao Yu was incorrect. This information has since been corrected in the published article. The corrected information may also be seen in the authorship of this erratum article. The publisher thanks you for reading this erratum and apologizes for any inconvenience caused.
